# A Digital-Health Program Based on Comprehensive Geriatric Assessment for the Management of Older People at Their Home: Final Recommendations from the MULTIPLAT_AGE Network Project

**DOI:** 10.3390/healthcare13101105

**Published:** 2025-05-09

**Authors:** Alberto Pilotto, Carolina Massone, Guido Iaccarino, Armando Genazzani, Carlo Trompetto, Gennarina Arabia, Wanda Morganti, Emanuele Seminerio, Maddalena Illario, Luigi Castello, Laura Mori, Loris Pignolo, Romina Custureri

**Affiliations:** 1 Geriatrics Unit, Department Geriatric Care, Orthogeriatrics and Rehabilitation, Galliera Hospital, 16128 Genoa, Italy; alberto.pilotto@galliera.it (A.P.); wanda.morganti@galliera.it (W.M.); emanuele.seminerio@galliera.it (E.S.);; 2 Department of Interdisciplinary Medicine, University of Bari “Aldo Moro”, 70121 Bari, Italy; 3 Department of Clinical Medicine and Surgery, University “Federico II” of Naples, 80138 Naples, Italy; 4 Department of Pharmaceutical Sciences, University of East Piedmont, 13100 Novara, Italy; 5 Department of Neuroscience, Rehabilitation, Ophthalmology, Genetics, Maternal and Child Health (DINOGMI), University of Genoa, 16126 Genoa, Italy; ctrompetto@neurologia.unige.it (C.T.);; 6 IRCCS Ospedale Policlinico San Martino, 16132 Genoa, Italy; 7 Neurologic Department, University of Catanzaro “Magna Graecia”, 88100 Catanzaro, Italy; 8 Public Health Department, University Federico II, 80138 Naples, Italy; illario@unina.it; 9 SSD Translational Medicine, Department of Integrated Research and Innovation Activities (DAIRI)-AOU of Alessandria, 15121 Alessandria, Italy; 10 Department of Traslational medicine, University of East Piedmont, 13100 Novara, Italy; 11 Institute “S. Anna”, 88900 Crotone, Italy

**Keywords:** aging in place, gerontechnology, Comprehensive Geriatric Assessment, Multidimensional Prognostic Index, personalized care, older people

## Abstract

Background: The MULTIPLAT_AGE is a network project which developed a digital platform based on the Comprehensive Geriatric Assessment (CGA) for collecting data and identifying personalized healthcare programs for older people at home. In this article, the final recommendations of the MULTIPLAT_AGE Working Group are reported. Methods: The MULTIPLAT_AGE project included five independent studies developed and carried out by five research centers according to two common principles previously shared by the researchers: (i) the multidimensional approach to older people through the CGA-based Multidimensional Prognostic Index (MPI); (ii) the use of a common web-based platform for collecting data to facilitate healthcare interventions of older people at their home according to the aging in place approach. At the end of the studies, a series of recommendations have been proposed by an expert panel including the principal investigators and discussed by all researchers involved in the MULTIPLAT_AGE project in formal meetings. After discussion, the recommendations have been approved with formal vote by all the researchers during the final meeting of the MULTIPLAT_AGE project. Results: The recommendations are addressed to healthcare providers, policy decision-makers, caregivers, and patients. In summary, the CGA-based interventions and technologies adopted in the MULTIPLAT_AGE project reduced length of hospital stay, improved multidimensional frailty, walking safety, physical and cognitive performances, and reduced fear of falling in older people across different clinical settings and suffering from different diseases. Conclusions: The final recommendations of the MULTIPLAT_AGE Working Group could be a useful instrument to facilitate the use of technologies along with CGA-based interventions to improve the management of older people at home.

## 1. Introduction

The worldwide increase in the aging population poses overwhelming pressure on the healthcare systems to develop innovative solutions to enable older people to maintain an overall good general health status and postpone dependency [1,2]. From this perspective, the concept of “aging in place”, which is the ability to live in one’s own home and community safely, independently, and comfortably, regardless of age, income, or ability level [2,3,4], could represent a new challenge related to the demographic changes. The literature reports significant benefits for older people living in their own homes, especially in psychological and emotional well-being [5] and functional aspects that may positively impact negative health outcomes including cognitive and functional decline, and risk and fear of falls [6,7].

Aging in place requires the presence of a network of integrated and coordinated healthcare services which are often lacking in real life [8]. The fragmented distribution of care, which is frequently provided in compartmentalized settings like hospitals, long-term care facilities, nursing homes, and home care services, makes it difficult to apply a comprehensive care plan in the real world [9], leading to inappropriate use of care services and higher healthcare expenses [10].

Several studies reported that care interventions based on a Comprehensive Geriatric Assessment (CGA) could improve health outcomes by reducing the fragmentation of care [9,10] and by addressing patient complexity [11,12]. Indeed, the CGA comprises a simultaneous evaluation of different domains such as functional, psychosocial, mobility, nutritional, and health status, especially focused on comorbidity and polypharmacy. CGA has already proved effective in reducing, across different settings and in different clinical conditions, negative outcomes such as hospitalization, admission to long-term care facilities, and preventing other adverse outcomes [13].

In this aforementioned context, the role of innovative technological solutions, such as Information and Communication Technology (ICT) systems, including telemedicine and teleassistance systems, domotics and robotics, could offer challenging opportunities for monitoring health status, supporting older people’s activities along with the strengthening of social connections [14,15]. Moreover, ICT could offer telecare and telerehabilitation systems, also allowing coordinated pathways of care across various settings and geographical areas [16].

The MULTIPLAT_AGE multicenter network project consisted of five independent studies; each study had its own main goal, as described below, and they shared some common characteristics: (i) the multidimensional approach to older people through the CGA-based Multidimensional Prognostic Index (MPI); (ii) a common web-based platform for collecting data to facilitate healthcare interventions of older people at their home according to the aging in place approach.

Findings of the five studies suggested that the CGA-based interventions and technologies used in the MULTIPLAT_AGE project reduced length of hospital stay, improved multidimensional frailty, walking safety, physical and cognitive performances, and reduced fear of falling in older people across different clinical settings and suffering from different diseases.

Given the background aforementioned, the aim of the present article is to describe the final recommendations as derived from the findings of the five studies of the MULTIPLAT_AGE project.

## 2. Materials and Methods

### 2.1. The MULTIPLAT_AGE Network Project

The MULTIPLAT_AGE project started in 2020 and ran until 2024, and consisted of five independent studies, performed in five research centers located in four Italian regions: Liguria, Campania, Calabria, and Piemonte. The general aims of the MULTIPLAT_AGE multicenter network project were to: (a) identify shared clinical and technological assessment tools and outcomes indicators across the different research units in order to approach the clinical complexity of community-dwelling multimorbid older adults; (b) develop a shared platform of multicomponent interventions able to guarantee a tailored approach to the heterogeneous needs of the multimorbid older adults; (c) share the multicomponent interventions strategy to improve the tailored care solutions to older subjects by enhancing synergy and complementarity among the different regions involved in the program.

Each individual study set its own specific objective: (1) the PRO–HOME study, developed by the Galliera Hospital of Genoa, aimed to design, develop, and implement a transitional care program (TCP) in a technologically equipped area to facilitate the discharge and reduce unnecessary prolonged length of hospital stay (LOS); (2) the EASYDOM study, developed by the Federico II University of Naples, aimed to design and test an ICT-based infrastructure promoting dietary habits, Adapted Physical Activity (APA), and treatment adherence; (3) the ORDER study, developed by the University Hospital “Maggiore della Carità” of Novara and the University of Piemonte Orientale in Novara, aimed to assess drug interactions in older adults, and the role of multidimensional frailty assessed by the Multidimensional Prognostic Index (MPI) in predicting mortality in older patients admitted to the Emergency Department (ED) for drug-related major bleeding; (4) the E-ACTION TRAINING study, developed by Ospedale Policlinico San Martino-IRCCS, aimed to evaluate feasibility and efficacy of an intervention based on Action Observation (AO) and physical exercises on balance and walking ability in older people at risk of falls; (5) the Stimo.TE-Rehab study, carried out by the University Hospital and University of Catanzaro and the Sant’Anna Hospital of Crotone, aimed to test the efficacy of a telerehabilitation-based Cognitive Stimulation (CS) treatment for improving cognitive performances and activities of daily living in patients with Parkinson’s Disease (PD) and chronic post-stroke impairment.

A brief description of the main objectives, methods, and results of the five studies of the MULTIPLAT_AGE project are reported in Table 1. Further details on inclusion and exclusion criteria are provided in Table A1 in Appendix A.

Despite the heterogeneity of the studies included in the MULTIPLAT_AGE project, they all shared the same target population, i.e., people aged 65 years and older, a common web-based platform for collecting data, and a standard evaluation based on the administration of a CGA with the computation of the MPI.

### 2.2. The Multidimensional Prognostic Index (MPI)

The Multidimensional Prognostic Index (MPI) is a validated CGA-based prognostic tool, demonstrating in many independent studies to be accurate to predict a variety of unfavorable health events, including mortality, risk of hospitalization, admission to long-term care facilities, and length of hospital stay in older adults.

The standard version of the MPI is computed using an algorithm drawing data from the following 8 domains:

1. Activities of Daily Living (ADL);
2. Instrumental Activities of Daily Living (IADL);
3. Short Portable Mental Status Questionnaire (SPMSQ);
4. Mini-Nutritional Assessment—Short Form (MNA-SF);
5. Exton-Smith Scale (ESS);
6. Cumulative Illness Rating Scale—comorbidity index (CIRS-CI);
7. Number of drugs;
8. Co-habitation status: in family, in institution, alone.

The total score for each domain is associated with a risk category score which must be summed and then divided by the number of completed domains (at least 6 to be computable) obtaining a continuous MPI total value between 0 and 1. Higher MPI scores indicate a higher risk. By using appropriate cutoff values, three classes of risk can be identified: low risk, 0.0–0.33; moderate risk, 0.34–0.66; and severe risk, 0.67–1.0.

The MPI was initially developed and validated in hospitalized older people; during the last 15 years, different versions of the MPI to facilitate the use of the MPI in different clinical settings, i.e., hospital wards, emergency department, general practice ambulatory, at population level, have been developed and validated. In the MULTIPLAT_AGE project, the following MPI version have been used:

- Standard Hospital Version [17,18,19];
- Outpatient Version: this version differs from the standard Hospital Version for the inclusion of the Barthel Mobility Scale instead of the ESS [23];
- TELE-MPI: telephone-administered version: the answers are simplified to facilitate remote communication [24,25];
- BRIEF-MPI: shorter version including just three relevant items for each domain explored [26].

### 2.3. The Digital Platform

**Figure 1:**
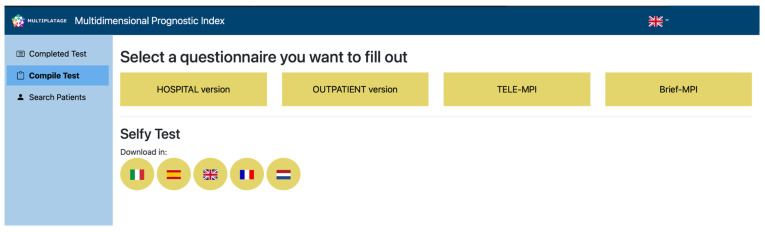
The MULTIPLAT_AGE digital platform.

The data collected from the five studies were gathered in an online platform (Figure 1) which included common and project-specific parameters. During the data collection and analysis phase, access to the platform was granted exclusively to the MULTIPLAT_AGE researchers.

At the end of the project, a website (https://multiplat-age.it/) (accessed on 11 March 2025) incorporating information on the overall program along with the single studies was made available with the purpose of disseminating contents and outcomes, and providing a permanent online space where the patient trajectories identified by the study may be consulted freely at any time.

### 2.4. Definition of the Recommendations

During the three-year period of the MULTIPLAT_AGE project, several online and on-site meetings were held to coordinate the overall project, and to lay the groundwork for the drawing of the recommendations.

The final results of each study were officially presented by the researchers and a panel of experts including the Principal Investigators of the five studies; MULTIPLAT_AGE was identified in a plenary meeting on 5 July 2024. During the following 5 months, the proposal of recommendations derived from the findings of the single studies were collected and discussed during two online meetings including participants of the expert panel and the researchers of the MULTIPLAT_AGE project.

From an organizational standpoint, the collection of evidence from each different working unit was supported by a framework that Principal Investigators (PIs) were asked to compile with summarized information about the methods and results of their study, along with a descriptive flowchart and a preliminary proposal of recommendations.

From an initial pool of 20 preliminary proposed recommendations, the members of the panel of experts evaluated consistency between recommendations and the statistically significant findings of the studies as well as the clinical and organizational potential usefulness for older people, caregivers, healthcare professionals, and policy decision-makers.

The panel of experts, composed of representatives from various fields and geographical areas, ensuring a wide interdisciplinary approach, reached consensus unanimously on the final recommendations focusing on: (i) safety of the technology employed; (ii) multidisciplinarity; (iii) clinical usefulness—both for the specific setting considered and for the path of care through the settings (i.e., from hospitalization to discharge and then to physical and cognitive rehabilitation activities).

Finally, 10 recommendations were selected based on the aforementioned criteria, i.e., two recommendations from each study. A formal vote took place during the final meeting held on 5 December 2024, reaching a plenary consensus on all 10 recommendations. Subsequently, a public event was held on 6 December 2024, to display the final recommendations to the audience.

## 3. Results

### MULTIPLAT_AGE Recommendations

Table 2 reports the recommendations as approved by the expert panel and the researchers of the MULTIPLAT_AGE project. Based on the findings of the five studies, two statements have been approved for each study for a total of ten recommendations. Recommendations focused on strategies and management solutions for personalized care pathways pursuing a multidisciplinary approach to counteract healthcare system fragmentation.

Below are the recommendations divided by individual study.

**Table 2:** Recommendations as approved by the expert panel and the researchers of the MULTIPLAT_AGE project.

**PRO-HOME Study**	In-hospital facility equipped with telemonitoring technologies and information collected through the CGA-based Multidimensional Prognostic Index (MPI) may allow the deployment of an efficient multidimensional transitional care program in hospitalized older patients. The domotic, robotic, and ICT-based monitoring program may reduce the length of hospital stay and improves multidimensional frailty, cognitive functions, and nutritional status after 6 months from hospital discharge.
**EASYDOM Study**	ICTs’ clinical monitoring may facilitate the conduction of APA programs at home guaranteeing safety, improving adherence to prescriptions, and reducing pre-frailty through ameliorating physical performance. ICTs at home may improve information exchange between healthcare professionals and patients along with the remote collection of data, in order to update the Electronic Health Record.
**ORDER Study**	ICTs are useful for identifying and monitoring polypharmacy and drug interactions further providing a real-time interactions list update, to improve prescriptive appropriateness and reduce the avoidable Adverse Drug Reactions (ADR) in polytreated older people. The use of CGA-based tools, such as the BRIEF-MPI and the TELE-MPI, is useful to predict short-term mortality and to assess multidimensional frailty in older people admitted to the ED with major bleeding.
**E-ACTION** **TRAINING** **Study**	Identifying frail subjects with balance impairments is relevant to programming a personalized multidomain intervention for improving rehabilitation outcomes. At-home telerehabilitation program based on physical exercises and Action Observation (AO) improves engagement and adherence to the rehabilitation intervention, increasing walking safety and reducing fear of falls.
**Stimo.TE-REHAB** **Study**	Cognitive telerehabilitation produces an improvement in cognitive and affective functions in patients with Parkinson’s Disease (PD) and with post-stroke consequences (PS) affected by mild/moderate cognitive impairments. Cognitive telerehabilitation may improve the caregiver’s burden.

## 4. Discussion

The MULTIPLAT_AGE multicenter network project is a remarkable and ambitious program aiming at sharing good clinical practices and providing recommendations for personalized care management pathways for older people. This network was among the first attempts in creating a common background for older people’s healthcare management based on the concept that an effective aging in place program requires a multidimensional and multidisciplinary approach to develop a CGA-based personalized multidomain and multicomponent intervention [13].

Indeed, although CGA-based interventions are already recognized as effective in reducing hospitalization, mortality, and institutionalization in older people in different settings [13], their application in conjunction with the use of technologies is still poorly studied. Moreover, technological devices can help older people, caregivers, and healthcare professionals in monitoring, assessing changes and rehabilitating, while keeping the patients at their home, as promoted by the “aging in place” concept. Indeed, previous studies showed that letting older people stay in their own environment is related to positive psychological and emotional outcomes [5], reduced risk of falls and related fear [27], along with other negative health outcomes such as functional impairments [7]. Despite the likely benefits of an aging in place approach, its practical application is uneven and hindered by the lack of shared guidelines aiming at tailoring home care to older people with variable degrees of multidimensional frailty and affected by different diseases. The findings of the five studies included in the MULTIPLAT_AGE multicenter network project converged in the drafting of the final recommendations, conceived to provide a guide to patients, as well as their caregivers, healthcare professionals, and policy decision-makers, displaying the efficacy and feasibility of managing older people at home with crucial support of robotics, domotics, and ICT devices in order to counteract the fragmentation of care.

MULTIPLAT_AGE highlighted how multidomain interventions using technological devices could be potentially feasible and safe in real-life healthcare management. The effectiveness of physical and cognitive telerehabilitation programs and transitional care programs based on telemonitoring in reducing unnecessarily prolonged LOS and in improving cognitive, affective, nutritional, the overall degree of multidimensional frailty, and gait-related subjective factors have been also proven.

ICT adoption enables the creation of a network connecting homes and clinical settings offering the possibility to continuously remotely exchange and share data among clinical staff, caregivers, and patients directly, allowing both the prompt intervention when significant changes occur or in case of ADR (relying on the updated Electronic Health Record) and avoiding, at the same time, unnecessary or deferrable visits.

Cognitive and physical rehabilitation and stimulation programs can effectively be conducted at home relying on different technologies, thus indirectly aiding caregivers in relieving the burden associated with care and organizational support [28] (i.e., hospital visits attendance) by bringing healthcare directly to people’s homes. Informal caregivers of older people, in fact, may suffer from caregiver burden due to the physical, financial, and psychosocial hardships of caring for another person [29]. This facilitation could improve adherence and engagement in rehabilitation programs or medical visits. The studies conducted within the MULTIPLAT_AGE project support these hypotheses as both APA prescriptions and physical rehabilitation showed increased adherence when held at home.

Finally, technological development granted the possibility to remotely assess the multidimensional profile of older people as demonstrated by the TELE-MPI which already proved as reliable as the in-person administered version [24], also confirming its prognostic value.

The MULTIPLAT_AGE network project has some limitations to account for. Even if the implementation of different technologies for each individual study—aimed at the deployment of domotic, robotic, and telecare systems—allowed the application of multidimensional interventions in many different settings, this heterogeneity could challenge the development of a shared ICT platform and the comparability of the collected data.

Another possible limitation is the still partial diffusion of CGA in clinical practice, aggravated by the diverse organization of each local health organization involved, which could hamper the deployment of the recommendations developed within the MULTIPLAT_AGE project. Moreover, the theoretical possibility and resources of carrying out a program or procedure often do not translate in its practical application, due, for instance, to a general lack of trained and dedicated personnel. Another aspect to be considered is that patient adherence and engagement are critical to the success of technology-assisted interventions. The overall efficacy of the suggested solutions may be impacted by variations in older individuals’ willingness or ability to interact with new technologies.

We have not standardized a set of questions to measure accessibility because technologies used were heterogeneous and study-specific, designed for different impairments, settings, and domains. However, we obtained information about acceptance and usability of the technology used by participants in the single studies. For example, in the EASYDOM and E-ACTION TRAINING studies, attention was dedicated to technology-related measures, respectively, about compliance and usability. In the PRO-HOME project, this assessment was carried out indirectly using a quality of life scale (the 12-item Short Form Survey–SF12) (see Table 1).

Moreover, while the aim of the MULTIPLAT_AGE project did not include formal cost-effectiveness analyses of technologies and methods adopted in the different studies, some indirect information about this has been reported in a specific study. For example, in the PRO-HOME study, the reduction of 2 days of length of hospital stay (LOS) in the intervention group compared to the control group could be an indicator of the potential cost-effectiveness improvement of this model. Further research will be needed to explore this important issue.

Additionally, the use of technology is still heavily influenced by socioeconomic factors such as income, education, and access to resources. Although the project was conducted entirely within the Italian healthcare system, which limits its generalizability to other contexts, this also ensures strong contextual relevance. In fact, it was a specific objective of the MULTIPLAT_AGE project (NET-2016-02361805) to connect different realities in different regions, involving the four different Italian regions that have a specific regional health organization.

The innovative contribution made by this study is certainly high and may prove to be an extremely useful starting point for future research.

Future efforts should focus on spreading the recommendations and best clinical practices developed within the MULTIPLAT_AGE network project at a regional and national level, along with the use of CGA in the different contexts and settings explored. Under this perspective, future research should explore the deployment of tailored interventions specifically designed for frail older populations, ensuring that care models are adaptable to diverse geographical and socioeconomic contexts and the fielding of appropriate training programs for healthcare professionals to improve CGA proficiency and digital literacy.

## Figures and Tables

**Table 1:** Main objectives, methods, and results of the five studies of the MULTIPLAT_AGE project.

STUDY	MAIN OBJECTIVE	METHODS	RESULTS
PRO-HOME	Testing the efficacy of a transitional care program based on a multicomponent intervention in reducing length of hospital stay (LOS) in older patients.	Randomized clinical trials on 60 hospitalized older patients. The intervention group (IG, n = 30) underwent a multicomponent intervention inside a technologically equipped protected discharge intra-hospital facility. The control group (CG, n = 30) underwent usual care until discharge. Variables: multidimensional frailty (MPI), Health-related Quality of Life (HRQOL), re-hospitalization, institutionalization, death rates. Follow-up: 1, 3, and 6 months.	In the IG, a 2-day reduction in LOS (*p* < 0.001), and improvement in multidimensional frailty (*p* = 0.040), i.e., nutritional (*p* = 0.002) and cognitive status (*p* = 0.041) were observed vs. CG. No changes in HRQOL, re-hospitalization, institutionalization, and death rates were observed in both groups. Only the CG showed a significant worsening in number of comorbidities (*p* = 0.014) [17,18,19].
EASYDOM	Testing the effectiveness of ICT-supported prescription and monitoring of patients with chronic conditions undergoing optimal treatment.	Randomized clinical trial on 102 hypertensive outpatients with physical pre-frailty (2 positive out of 5 Fried Criteria) treated with Adapted Physical Activity (APA). Two groups involved: Intervention group (IG, n = 50): APA prescription plus ICT support kit; Control group (CG, n = 52): receiving only APA prescription (no ICT support kit). Variables: Reduction in cardiovascular risk, according to the Italian Institute of Health risk maps, number of unplanned hospitalizations, physical activity, and fitness parameters. Follow-up: 6 months.	The ICT-supported Adapted Physical Activity (APA) program led to greater improvements in physical performance in IG compared to the CG. Participants in the IG showed a significant increase in exercise tolerance after the 3-month intervention. After 6 months of follow-up, a decrease in the pre-frailty incidence compared to baseline in all groups was observed: the decrease was greater in the IG vs. CG.
ORDER	A. To assess the prevalence of polypharmacy and drug interactions in community-dwelling older adults. B. To assess the accuracy of the BRIEF-MPI to predict short-term mortality in older adults admitted to the ED for a major bleeding.	A. Retrospective observational cohort study (study period from the year 2013 to 2019) of over a million over-65 residents in the Piedmont Regional Health Data Warehouse (CLONE): over a period of 6 years. Variables: polypharmacy and drug interaction rates. Follow up: not applicable per study design. B. Prospective observational cohort study on 62 older patients admitted to the ED for major bleeding. Variables: multidimensional frailty (according to BRIEF-MPI and TELE-MPI), mortality rates. Follow-up: 24 h, 48 h, and 1 month after ED admission.	A. 15% of the over-65 population was polytreated (more than 5 drugs prescribed in the same solar year) but a significant reduction in the prevalence of drug interactions compared to what was reported in the previous decade was observed. Furthermore, a decline in drug interactions was also observed in the 6-year span covered by the study [20]. B. The BRIEF-MPI showed a good accuracy in predicting short-term mortality (*p* < 0.001) in older patients admitted to the ED for major bleeding: Area Under the Curve (AUC) = 0.753; in patient survivors a significant reduction of multidimensional frailty as assessed by the TELE-MPI (*p* < 0.017) after 1 month.
E-ACTION TRAINING	Evaluating the efficacy of a home-based training, combining Action Observation (AO) and physical exercises (E-ACTION TRAINING) to improve balance and gait performances in people at risk of falls.	Comparative study with a pre-post design conducted on 60 patients, divided into three groups (20 participants per group): Healthy older adults (aged 70–85 years). Patients with chronic stroke (aged 60–85 years), able to walk independently. Patients with Parkinson’s Disease (PD) (aged 60–85 years) at Hoehn and Yahr stages II–III. Variables: (1) to assess balance and gait, including: 2-Minute Walk Test (2MWT), Short Physical Performance Battery (SPPB), Timed Up and Go Test (TUG), Four Square Step Test (FSST), Mini-Balance Evaluation Systems Test (Mini-BESTest), Activities specific Balance Confidence Scale (ABC Scale), Montreal Cognitive Assessment (MoCA); (2) to assess quality of life: SF-36 Health Survey 3) to assess usability: System Usability Score (SUS). Assessment: before and after the training period.	Patients in all three groups showed a significant improvement in several outcome measures assessing balance and gait after the treatment. However, only the healthy older adults group also exhibited an improvement in quality of life. System Usability Score showed excellent performance in all the three groups.
Stimo.TE-REHAB	Evaluating the effectiveness of Remote Cognitive Stimulation (RCS) in improving cognitive performances and activities of daily living.	Randomized controlled trial on 81 older patients with mild to moderate cognitive impairment, assigned to RCS Stimo.TE-Rehab (intervention group, IG) or traditional Cognitive Stimulation (control group, CG). The total sample included: Parkinson’s Disease patients (PD, n = 45); post-stroke patients (PS, n = 36). Variables: visuospatial abilities (VSA), attention and working memory, language and executive functions, mood (M) as assessed by Beck Depression Inventory, BDI + State Trait Anxiety Inventory (STAI), Short Form 36 Health Outcome (SF-36), caregiver burden (CB). Assessment: before and after the treatment. Follow-up: 6 months after treatment.	Stimo.TE-Rehab IG: patients with PD increased their VSA (*p* = 0.014), attention and working memory (*p* = 0.046), and language and executive functions (*p* = 0.037); patients with PS showed a significant improvement in language (*p* = 0.001), M (*p* = 0.03), and showed a reduction in CB (*p* = 0.04). Stimo.TE-Rehab CG: patients with PD showed an improvement in VSA (*p* = 0.014), language (*p* = 0.010), and M (*p* = 0.048); patients with PS showed a significant improvement in VSA (*p* = 0.04), attention, and executive functions (*p* = 0.01) [21,22].

## Data Availability

The data are available upon request from the corresponding author.

## Appendix A

**Table A1:** Inclusion and exclusion criteria of the five studies of the MULTIPLAT_AGE project.

STUDY	INCLUSION CRITERIA	EXCLUSION CRITERIA
PRO-HOME	Patients aged ≥65 years admitted to the Geriatrics Unit for an acute event, deemed stable and dischargeable; Complete personal autonomy or with mild to moderate limitation (ADL ≥ 3/6); Cognitively intact or mild–moderate cognitive impairment (SPMSQ ≤ 5/10); Informed consent signed;	Clinical instability; Behavioral disturbances and/or uncontrolled psychotic symptoms; Severe limitation of personal autonomy (ADL < 3/6); Severe cognitive impairment (SPMSQ > 5/10); Lack of informed consent.
EASYDOM	Patients aged ≥60 years; Patients with moderate cardiovascular risk; Patients referred to the adapted physical activity prescription clinic; Patients with chronic diseases and multimorbidities.	Neoplasm; Life expectancy less than 24 months; Multidimensional Prognostic Index >0.67; Digital technology illiterates.
ORDER	A. Community dwelling people aged ≥65 years; Treated with polypharmacy (≥5 concomitant drugs taken for at least 90 days in the first 6 months of the year). B. Patients on anticoagulant therapy (Vitamin K Antagonists—VKAs or Direct Oral AntiCoagulants—DOACs); Admitted to the ED for major bleeding; Aged ≥65 years.	A. Patients aged <65 years; Patients not resident in the Piedmont Region. B. Admitted to the ED for other problems; Aged <65 years.
E-ACTION TRAINING	Common: 2 or more falls within 6 months prior to enrolment; Able to walk for 5 min unassisted; Adequate hearing (as evaluated by the whisper test) and vision capabilities (as measured using a Snellen chart); Stable medication for the past 1 month and anticipated over a period of 6 months. Specific for healthy subjects: Aged 70–85. Specific for PD subjects: Aged 65–85; Diagnosis of idiopathic PD, as defined by the U.K. Brain Bank criteria Hoehn and Yahr stage II–III; Taking anti-Parkinsonian medication; Stable medication for past 1 month before enrolment and anticipated over next 6 months. Specific for post-stroke subjects: Aged 65–85; Stroke diagnosis, supported by neuro-images (MRI or TC); More than 6 months after stroke onset (chronic phase);	Common: Psychiatric comorbidity (e.g., major depressive disorder as determined by DSM IV criteria); Clinical diagnosis of dementia or other severe cognitive impairment (Mini-Mental State Examination score <24); History of traumatic brain injury or other neurological disorders (other than PD and stroke, for those groups). Unstable medical condition including cardiovascular instability in the past 6 months; Currently participating in another interfering therapy or in another trial. Specific for healthy subjects: Aged <70 or >85. Specific for PD subjects: Severe freezing of gait (defined as having >15 on the new FOG questionnaire); Deep Brain Stimulation; Duodenal infusion pump. Specific for post-stroke subjects: Significant aphasia;
Stimo.TE-REHAB	Diagnosis of ischemic stroke in the territory of the middle or anterior cerebral artery; Aged ≥65; Presence of mild motor impairment with persistent cognitive deficits; clinically stable condition; Time since stroke ≥8 months; Absence of post-stroke complications (e.g., infections, seizures); Eligibility for receiving in-home neurorehabilitation services.	Presence of other non-vascular brain lesions; History of dementia or psychiatric disorders; History of regular prior and/or current substance (drug/alcohol) abuse; Absence of cognitive impairment (Mini-Mental State Examination (MMSE) score >24); Aphasia, as assessed by the Aachener Aphasie Test (AAT); Severe visual impairment.
